# Adoption, Perceived Barriers, and Future Perspectives of Guided Endodontics for the Management of Calcified Root Canals Among Endodontists: A Cross-Sectional Questionnaire-Based Study

**DOI:** 10.7759/cureus.115637

**Published:** 2026-09-02

**Authors:** Mohd Zeeshan Ahmad, Krishna P Biswas, Diwakar Vasisth, Aarti S Bedia, Sanpreet S Sachdev, Anil Richhawal

**Affiliations:** 1 Department of Conservative Dentistry and Endodontics, Rama Dental College Hospital and Research Centre, Kanpur, IND; 2 Department of Dentistry, All India Institute of Medical Sciences, Guwahati, Guwahati, IND; 3 Department of Conservative Dentistry and Endodontics, Pro Healthcare, New Delhi, IND; 4 Department of Oral Medicine and Radiology, Bharati Vidyapeeth (Deemed to be University) Dental College and Hospital, Navi Mumbai, IND; 5 Department of Oral Pathology and Microbiology, Bharati Vidyapeeth (Deemed to be University) Dental College and Hospital, Navi Mumbai, IND; 6 Department of Conservative Dentistry and Endodontics, Dr. G.D. Pol Foundation Y.M.T. Dental College and Hospital, Navi Mumbai, IND

**Keywords:** cone-beam computed tomography, endodontics, root canal calcification, surveys and questionnaires, three-dimensional printing

## Abstract

Introduction: Guided endodontics has emerged as a promising digital approach for the management of calcified root canals by improving the accuracy of canal localization and minimizing procedural complications. Despite its clinical advantages, information regarding its adoption and factors influencing its implementation among endodontists remains limited. This study aimed to evaluate the adoption of guided endodontics for managing calcified root canals, identify perceived barriers to its implementation, and assess clinician perspectives regarding its future integration into routine endodontic practice.

Materials and methods: A cross-sectional questionnaire-based survey was conducted among practicing endodontists over a four-month period. A validated, self-administered questionnaire comprising 12 questions distributed across four sections was used to evaluate demographic characteristics, clinical application of guided endodontics, perceived barriers, and future perspectives. The questionnaire was subjected to expert validation, pilot testing, and a reliability assessment before distribution. Responses were recorded on a five-point Likert scale. Data were analyzed statistically using Cronbach's alpha, the Shapiro-Wilk test, the Mann-Whitney U test, the Kruskal-Wallis H test, and the chi-square test, with statistical significance set at p < 0.05.

Results: In total, 290 completed questionnaires were analyzed. Formal training in guided endodontics was reported by 140 (48.3%) participants, while 150 (51.7%) had no formal training. The questionnaire demonstrated good internal consistency, with Cronbach's alpha values of 0.88 for clinical application, 0.85 for perceived barriers, and 0.86 for solutions and future outlook. Greater clinical experience was significantly associated with higher clinical application scores (p = 0.027) and more favorable solutions and future outlook scores (p = 0.009), whereas less experienced clinicians reported significantly greater perceived barriers (p = 0.018). Academic and public sector practitioners perceived significantly greater barriers than private practitioners (p < 0.001). Endodontists with formal training demonstrated significantly higher clinical application scores (3.9 ± 0.8 vs. 3.2 ± 0.9; p = 0.001) and significantly lower perceived barriers (3.2 ± 0.8 vs. 3.9 ± 0.8; p < 0.001) than those without formal training. Routine clinical use of guided endodontics was reported by 75 (53.6%) formally trained clinicians compared to 47 (31.3%) without formal training (χ² = 14.4, p = 0.001).

Conclusions: Guided endodontics is widely perceived as a valuable technique for the management of calcified root canals. However, its routine clinical implementation is influenced by clinician training, experience, and resource availability. Expanding structured educational programs and improving access to digital technologies may facilitate the greater adoption of guided endodontics and enhance the quality and predictability of endodontic care.

## Introduction

Calcification of the root canal system is a common consequence of aging, trauma, restorative procedures, and long-standing pulpal irritation, often resulting in partial or complete obliteration of the canal space [1]. These cases present one of the greatest technical challenges in endodontic practice because canal localization becomes highly unpredictable, increasing the risk of excessive dentin removal, perforation, instrument deviation, and eventual treatment failure [2]. Successful management of calcified canals requires accurate diagnosis, meticulous treatment planning, and advanced clinical skills, making these procedures considerably more demanding than conventional endodontic treatments [3]. Several conventional and contemporary approaches are available for the management of calcified root canals besides guided endodontics. These include magnification-assisted canal localization, ultrasonic troughing, the use of small pre-curved hand files with appropriate chelating agents, and cone-beam computed tomography (CBCT)- assisted conventional access and canal negotiation. Dynamic navigation represents another technology-assisted approach that provides real-time guidance during canal localization. When orthograde negotiation is unsuccessful or contraindicated, surgical endodontic approaches may also be considered. The choice of technique depends on the extent and location of canal calcification, tooth anatomy, available imaging and equipment, and operator expertise [1-3].

Recent advances in digital dentistry have introduced guided endodontics as an innovative approach to the management of calcified canals [4]. Guided endodontics combines CBCT, intraoral or laboratory optical scanning, three-dimensional planning software, and additive manufacturing to fabricate patient-specific guides that facilitate precise access cavity preparation [5]. This technology enables clinicians to locate obliterated canals with greater accuracy while preserving healthy tooth structures and minimizing iatrogenic complications [6]. Several in vitro and clinical investigations have demonstrated that guided endodontics improves the predictability of canal negotiation, reduces procedural errors, shortens treatment time, and enhances overall treatment outcomes, particularly in teeth with severe pulp canal obliteration [6,7].

Despite these advantages, the widespread implementation of guided endodontics in routine clinical practice remains limited [4]. The adoption of this technology is influenced by several factors, including the availability of CBCT imaging, access to digital planning software and three-dimensional printing facilities, treatment costs, operator training, and familiarity with the digital workflow [4]. Furthermore, variations in clinical experience, practice settings, and educational exposure may substantially influence clinicians' perceptions of its usefulness and feasibility. Although the literature has extensively evaluated the technical accuracy and clinical outcomes of guided endodontics, relatively little attention has been directed toward understanding the perspectives of practicing endodontists regarding their clinical application, barriers to implementation, and future adoption [4-6].

Therefore, the present cross-sectional questionnaire-based study was conducted to evaluate the adoption of guided endodontics for the management of calcified root canals among endodontists and to assess their perceptions regarding its clinical utility, barriers to implementation, and future prospects. Additionally, this study aimed to determine whether clinician characteristics, including years of clinical experience, practice setting, and formal training in guided endodontics, were associated with technology adoption and attitudes toward its integration into contemporary endodontic practice.

## Materials and methods

This cross-sectional questionnaire-based study was conducted in the Department of Conservative Dentistry and Endodontics, Bharati Vidyapeeth (Deemed to be University) Dental College and Hospital, Navi Mumbai, Maharashtra, India, after obtaining approval from the Institutional Ethics Committee (BVDUDC&H-IEC-360/2022 dated August 12, 2022) of the college. The study was conducted over a four-month period from September 2022 to December 2022 in accordance with the ethical principles of the Declaration of Helsinki for research involving human participants. Participation in the survey was voluntary, and informed consent was obtained electronically from all participants prior to the completion of the questionnaire. Confidentiality and anonymity of the respondents were maintained throughout the study, and no personally identifiable information was collected.

A structured self-administered questionnaire was specifically developed to assess the adoption of guided endodontics for the management of calcified root canals, perceived barriers to its implementation, and future perspectives among endodontists. The questionnaire was prepared by a multidisciplinary expert panel comprising specialists in conservative dentistry and endodontics and oral medicine and radiology from different dental institutions to ensure comprehensive evaluation of the study objectives and to incorporate expert opinions from multiple specialties. Following the initial draft, the questionnaire was validated by an expert panel to assess the relevance, clarity, comprehensiveness, and appropriateness of each item. Based on the experts' recommendations, minor modifications in the wording and sequencing of questions were made to improve readability and eliminate ambiguity.

The final questionnaire was pilot-tested among 50 endodontists who were not included in the final analysis to evaluate its feasibility, comprehensibility, and completion time. Feedback obtained during the pilot testing was incorporated before the commencement of the main survey. The internal consistency reliability of the questionnaire was subsequently assessed using Cronbach's alpha coefficient, which demonstrated good reliability for all domains (α > 0.80), confirming the satisfactory internal consistency of the instrument. The final questionnaire consisted of 12 numbered questions organized into four sections. Section A included three demographic and professional questions (Q1-Q3). Sections B, C, and D comprised 14, 12, and nine Likert-scale items, respectively, assessing clinical application, perceived barriers, solutions, and future perspectives (Appendix 1). Section A included three questions on demographic and professional characteristics, namely, years of clinical experience, practice setting, and previous training in guided endodontics. Section B assessed the clinical application of guided endodontics using four questions evaluating clinical utility, frequency of use, diagnostic and treatment planning approaches, and perceived clinical outcomes. Section C comprised three questions exploring perceived barriers related to financial constraints, infrastructure, technical expertise, training, and workflow limitations. Section D contained two questions evaluating potential solutions and future perspectives regarding guided endodontics, including educational needs, technological advancements, institutional support, and anticipated future adoption. Responses to Sections B, C, and D were recorded using a five-point Likert scale ranging from one (strongly disagree) to five (strongly agree), with higher scores indicating greater agreement with the respective statements.

The questionnaire was converted into an electronic survey using Google Forms (Google LLC, Mountain View, California, United States) and distributed to practicing endodontists across India through professional associations, institutional mailing lists, and social media platforms, including WhatsApp (Meta Platforms, Inc., Menlo Park, California, United States) and email. Reminder messages were circulated periodically during the study period to improve the response rate. Only completed questionnaires submitted by qualified endodontists were included in the final analysis; duplicate or incomplete responses were excluded.

The sample size was estimated using Calculator.net based on pilot data obtained from 50 endodontists, of which approximately 20% reported routine utilization of guided endodontics for managing calcified canals. Assuming a 95% confidence level, a 5% absolute margin of error, and accounting for an anticipated 20% non-response rate, the minimum required sample size was calculated as 296 participants and rounded to a target sample of 300 endodontists for the survey distribution. Ultimately, 290 complete responses were received and included in the statistical analysis. The 50 endodontists who participated in the pilot study were excluded from the final survey sample to avoid data overlap and ensure the independence of the primary analytical dataset.

Statistical analyses were performed using IBM SPSS Statistics for Windows, Version 25 (Released 2017; IBM Corp., Armonk, New York, United States). Descriptive statistics, including frequencies, percentages, means, and standard deviations, were used to summarize the participant characteristics and questionnaire responses. The internal consistency reliability of the multi-item domains was evaluated using Cronbach's alpha. The Shapiro-Wilk test was applied to assess data normality, following which appropriate non-parametric statistical tests were employed. Comparisons of domain scores among groups based on years of clinical experience and practice settings were performed using the Kruskal-Wallis H test with corresponding effect sizes (η²), whereas comparisons according to formal training status were conducted using the Mann-Whitney U test with rank-biserial effect sizes. Post hoc analysis was done using the Dunn test. Associations between categorical variables, including formal training and routine clinical use of guided endodontics, were analyzed using the chi-square test, with Cramér's V as the measure of effect size. All statistical tests were two-tailed, and statistical significance was set at p < 0.05.

## Results

In total, 290 endodontists completed the questionnaire and were included in the final analysis. The demographic and professional characteristics of the participants are summarized in Table 1. Around 90 (31%) respondents had five to 10 years of clinical experience, followed by 80 (27.6%) respondents with 11-20 years of clinical experience. Most participants engaged in private solo practice, while 80 (27.6%) practiced in private group settings. Formal training in guided endodontics was reported by 140 (48.3%) participants, including hands-on training by 80 (27.6%) and theoretical training by 60 (20.7%), whereas 150 (51.7%) had no formal training or were self-learned (Table 1).

**Table 1:** Demographic and professional characteristics of the study participants. Data are presented as n (%), and percentages were calculated based on the total study population (N = 290).

Characteristic	Category	n (%)
Experience	<5 years	60 (20.7)
	5–10 years	90 (31.0)
	11–20 years	80 (27.6)
	>20 years	60 (20.7)
Formal training	Hands‑on	80 (27.6)
	Theoretical only	60 (20.7)
	Self‑learned	70 (24.1)
	None	80 (27.6)
Practice setting	Private solo	100 (34.5)
	Private group	80 (27.6)
	Academic / University	60 (20.7)
	Government / Public hospital	30 (10.3)
	Corporate dental chain	20 (6.9)

The questionnaire demonstrated good internal consistency across all the evaluated domains (Table 2). The clinical application domain showed a Cronbach's alpha of 0.88, followed by the solutions and future outlook domain (0.86) and the perceived barriers domain (0.85), indicating satisfactory reliability of the questionnaire.

**Table 2:** Internal consistency reliability of the questionnaire domains. Data are presented as mean ± standard deviation (SD) unless otherwise indicated, internal consistency was assessed using Cronbach's alpha coefficient, skewness values describe data distribution, α: Cronbach's alpha.

Domain	Number of items	Cronbach’s α	Mean ± SD	Skewness
Clinical application	14	0.88	3.5 ± 0.9	−0.4
Perceived barriers	12	0.85	3.6 ± 0.8	−0.2
Solutions and future outlook	9	0.86	4.2 ± 0.7	−0.7

Clinical experience significantly influenced participants' perceptions (Table 3). Endodontists with more clinical experience demonstrated significantly higher clinical application scores (p = 0.027) and more favorable solutions and future outlook scores (p = 0.009), whereas perceived barriers were significantly higher among less experienced clinicians (p = 0.018).

**Table 3:** Comparison of questionnaire domain scores according to years of clinical experience (N = 290). Data are presented as mean ± standard deviation (SD), comparisons were performed using the Kruskal-Wallis H test, effect size is expressed as eta-squared (η²), *p < 0.05 was considered statistically significant, df: degrees of freedom, η²: eta-squared.

Domain	< 5 yrs (n = 60)	5–10 yrs (n = 90)	11–20 yrs (n = 80)	> 20 yrs (n = 60)	H‑value	df	p‑value	Effect size (η²)
Clinical application	3.2 ± 0.9	3.4 ± 0.9	3.7 ± 0.8	3.8 ± 0.8	9.2	3	0.027*	0.064
Perceived barriers	3.9 ± 0.8	3.8 ± 0.7	3.4 ± 0.8	3.3 ± 0.8	10.1	3	0.018*	0.070
Solutions and future outlook	4.1 ± 0.7	4.2 ± 0.6	4.3 ± 0.6	4.5 ± 0.5	11.5	3	0.009*	0.080

Post hoc comparisons using the Dunn test revealed that endodontists with >20 years of experience had significantly higher clinical application scores than those with <5 years (mean difference = -0.60, p = 0.045). Regarding perceived barriers, the <5 years group reported significantly greater barriers than both the 11-20 years (mean diff = 0.50, p = 0.048) and >20 years (mean diff = 0.60, p = 0.015) groups; similarly, the five to 10 years group reported greater barriers than the >20 years group (mean diff = 0.50, p = 0.042). No significant pairwise differences were observed for the solutions and future outlook domain (Table 4).

**Table 4:** Pairwise comparison of groups for clinical experience using post-hoc Dunn test with Bonferroni corrections. A: <5 years, B: 5–10 years, C: 11–20 years and D: >20 years, *p < 0.05 was considered statistically significant.

Comparison	Clinical application			Perceived barriers			Solutions and future outlook		
	Mean difference	Test statistic (q)	p-value	Mean difference	Test statistic (q)	p-value	Mean difference	Test statistic (q)	p-value
A vs. B	-0.2	1.407	0.853	0.1	0.779	0.963	-0.1	0.995	0.902
A vs. C	-0.5	3.431	0.085	0.5	3.799	0.048*	-0.2	1.940	0.684
A vs. D	-0.6	3.851	0.045*	0.6	4.267	0.015*	-0.4	3.630	0.056
B vs. C	-0.3	2.288	0.521	0.4	3.378	0.092	-0.1	1.079	0.883
B vs. D	-0.4	2.813	0.282	0.5	3.893	0.042*	-0.3	2.985	0.180
C vs. D	-0.1	0.686	0.971	0.1	0.760	0.965	-0.2	1.940	0.684

A comparison according to practice setting revealed a significant difference only in the perceived barriers domain (Table 5). Endodontists working in academic and public institutions reported significantly greater barriers than those working in private practice (p < 0.001). No significant differences were observed in clinical application (p = 0.14), solutions, and future outlook (p = 0.21) among the different practice settings. Post hoc Dunn test showed significant pairwise differences for the perceived barrier domain.

**Table 5:** Comparison of questionnaire domain scores according to practice setting (N = 290). Data are presented as mean ± standard deviation (SD), comparisons were performed using the Kruskal-Wallis H test, effect size is expressed as eta-squared (η²), *p < 0.05 was considered statistically significant, df: degrees of freedom, η²: eta-squared.

Domain	Private practice (n = 180)	Academic/Public (n = 90)	Corporate chain (n = 20)	H‑value	df	p‑value	Effect size (η²)
Clinical application	3.6 ± 0.9	3.4 ± 0.9	3.3 ± 1.0	4.0	2	0.14	0.028
Perceived barriers	3.3 ± 0.8	4.2 ± 0.7	3.8 ± 0.9	15.2	2	< 0.001*	0.105
Solutions and future outlook	4.2 ± 0.7	4.3 ± 0.7	4.1 ± 0.8	3.1	2	0.21	0.021

Formal training in guided endodontics was significantly associated with greater clinical adoption and fewer perceived barriers (Table 6). Endodontists who had received formal training demonstrated significantly higher clinical application scores (p = 0.001) and significantly lower perceived barriers (p < 0.001) than those who had not received formal training. However, no significant difference was observed in the solutions and future outlook domains between the two groups (p = 0.35).

**Table 6:** Comparison of questionnaire domain scores according to formal training in guided endodontics (N = 290). Data are presented as mean ± standard deviation (SD), comparisons were performed using the Mann-Whitney U test, effect size is expressed as rank-biserial correlation (r), *p < 0.05 was considered statistically significant, r: rank-biserial correlation.

Domain	Formally trained (n = 140)	No formal training (n = 150)	U‑value	p‑value	Effect size (r)
Clinical application	3.9 ± 0.8	3.2 ± 0.9	1750	0.001*	0.27
Perceived barriers	3.2 ± 0.8	3.9 ± 0.8	1680	< 0.001*	0.29
Solutions and future outlook	4.3 ± 0.7	4.2 ± 0.7	2400	0.35	0.08

A significant association was observed between formal training and routine clinical use of guided endodontics (Table 7). Among formally trained endodontists, 75 (53.6%) reported routine use of guided endodontics compared to 47 (31.3%) without formal training. This association was statistically significant (χ² = 14.4, p = 0.001), indicating that formal training was positively associated with the routine clinical implementation of guided endodontics.

**Table 7:** Association between formal training and routine clinical use of guided endodontics (N = 290). Data are presented as n (%), associations were analyzed using the chi-square test, effect size is expressed as Cramér's V, routine clinical use was defined as a score of ≥4 (agree/strongly agree) on questionnaire item Q5a, *p < 0.05 was considered statistically significant, χ²: chi-square statistic.

Training status	Routine users, n (%)	Non‑routine users, n (%)	Total, n (%)	Chi‑square (χ²)	p‑value	Effect size (Cramér’s V)
Formally trained (n=140)	75 (53.6)	65 (46.4)	140 (100.0)	14.4	0.001*	0.22
No Formal training (n=150)	47 (31.3)	103 (68.7)	150 (100.0)			
Total	122 (42.1)	168 (57.9)	290 (100.0)			

## Discussion

The findings of our study demonstrated that although endodontists generally recognized the clinical value of guided endodontics, its routine implementation remained moderate and was significantly influenced by formal training, years of clinical experience, and the practice setting. These observations highlight the gap between the awareness of digital technologies and their translation into routine clinical practice. Calcified canals continue to represent one of the most technically demanding scenarios in endodontics because conventional access preparation is associated with an increased risk of perforation, excessive dentin removal, and failure to locate the canal [1]. Guided endodontics has emerged as a predictable alternative that integrates CBCT imaging, digital planning, and three-dimensional printing to enable minimally invasive and accurate access cavity preparation [4]. Previous laboratory and clinical investigations by Krastl et al. [8], Connert et al. [9], and Zehnder et al. [10] demonstrated significantly greater accuracy, improved preservation of tooth structure, and reduced procedural errors with guided endodontics than with conventional techniques. The present findings are consistent with these reports, as respondents generally perceived guided endodontics as improving clinical predictability and treatment outcomes, indicating that clinicians acknowledge their potential benefits despite limited routine utilization.

An important finding of this study was the significant association between clinical experience and technology adoption. Endodontists with greater clinical experience demonstrated higher clinical application scores and more optimistic perceptions regarding the future of guided endodontics, whereas less experienced clinicians perceived significantly greater barriers. This finding is supported by previous literature [11,12]. Similar observations have been reported in surveys evaluating the adoption of digital technologies in dentistry, where experienced clinicians often demonstrate greater confidence in integrating advanced technologies once adequate training and clinical exposure have been achieved [13,14]. Conversely, younger clinicians may be more familiar with digital workflows but frequently encounter financial and infrastructural constraints during the early stages of practice.

The practice settings also influenced the participants' perceptions. Endodontists working in academic institutions and public hospitals reported significantly greater barriers than those working in private practice. This finding may reflect limited institutional resources, restricted access to CBCT units, digital planning software, three-dimensional printing facilities, and administrative delays associated with the procurement of advanced technologies [15]. Similar challenges have been reported in previous surveys investigating digital dentistry implementation, where infrastructure and equipment availability were identified as the major determinants of technology adoption [14,15]. Interestingly, despite these differences, clinicians across all practice settings expressed similar positive expectations regarding the future role of guided endodontics, suggesting widespread recognition of its clinical potential.

Perhaps the most clinically relevant finding of the present study is the influence of formal training. Endodontists who received structured hands-on or theoretical training reported significantly greater clinical adoption and significantly fewer perceived barriers than those who did not. Furthermore, formally trained clinicians are more likely to routinely use guided endodontics in clinical practice. These findings agree with previous studies demonstrating that continuing professional education and hands-on training substantially improves clinician confidence and facilitates the incorporation of digital workflows into routine practice [16-18]. The results emphasize that inadequate training remains a modifiable barrier, and that educational interventions may be more effective than technological advancements alone in improving clinical implementation.

Although respondents recognized several barriers, including treatment cost, digital workflow complexity, infrastructure requirements, and the need for additional training, nearly all participants expressed optimism regarding the future of guided endodontics [19]. The strong agreement for increased educational opportunities, improved software usability, affordable digital infrastructure, and institutional support suggests that clinicians anticipate wider adoption as digital technologies become more accessible [16]. With the increasing integration of artificial intelligence, automated treatment planning, and chairside three-dimensional printing, guided endodontics is expected to become more efficient and economically feasible in the coming years [20].

The clinical implications of this study are significant. The findings demonstrate that expanding access to structured training programs, incorporating guided endodontics into postgraduate curricula, and improving the availability of affordable digital infrastructure may substantially increase the adoption of guided endodontics [18]. Professional organizations, universities, and industry partners should collaborate to provide continuing education programs that enhance clinician competence and confidence in digital endodontic workflows [17]. Greater accessibility to CBCT imaging, computer-aided design software, and three-dimensional printing technologies may ultimately improve treatment predictability and patient outcomes for teeth with calcified canals [13].

This study has several limitations. The cross-sectional design precludes establishing causal relationships between clinician characteristics and adoption of guided endodontics. The use of an online, self-administered, self-reported questionnaire may have introduced recall and social-desirability bias. Furthermore, participants were recruited through professional associations, institutional mailing lists, and social media platforms, representing a non-probability sampling approach that may have introduced selection and response bias and may limit the generalizability of the findings to the wider population of endodontists in India. Although participants were recruited from across India, the final sample comprised 290 completed responses, which was slightly below the initially calculated minimum sample size of 296. This modest shortfall may have reduced the statistical power to detect smaller differences or associations.

Future studies should include multicenter and international surveys involving larger and more diverse populations to validate these findings. Longitudinal studies evaluating changes in adoption following structured educational interventions would provide stronger evidence of the impact of training. Furthermore, future investigations should explore cost-effectiveness, patient-reported outcomes, and the integration of emerging technologies, such as artificial intelligence-assisted planning, dynamic navigation, and augmented reality, to further optimize guided endodontic treatment [21].

## Conclusions

Guided endodontics is widely recognized by endodontists as a valuable technique for managing calcified root canals; however, its routine clinical adoption remains influenced by formal training, clinical experience, and resource availability. Structured education and improved access to digital technologies may enhance clinician confidence and implementation. Addressing financial, infrastructural, and educational barriers may facilitate broader integration of guided endodontics into contemporary endodontic practice and may support more predictable management of calcified root canals, although the present study did not directly evaluate treatment or patient outcomes.

## Appendices

Appendix 1

**Table 8:** Questionnaire design for the study.

SECTION A: Demographic and professional profile	
Q1. How many years of clinical experience do you have as an endodontist?	Less than 5 years
	5–10 years
	11–20 years
	More than 20 years
Q2. What is your primary practice setting?	Private practice (solo)
	Private practice (group/multispecialty)
	Academic institution / University hospital
	Government / Public hospital
	Corporate dental chain
Q3. Have you received any formal training or continuing education in guided endodontics?	Yes, hands-on training
	Yes, theoretical training only (lectures/webinars)
	No formal training, but self-learned
	No training at all
SECTION B: Clinical application of guided endodontics for calcified canals (Please rate your agreement with each statement on a scale of 1–5: 1=Strongly Disagree, 2=Disagree, 3=Neutral, 4=Agree, 5=Strongly Agree).	
Q4. Clinical utility of guided endodontics for calcified canals	
a. Guided endodontics is clinically useful for locating calcified root canals	□ Strongly Disagree, □ Disagree, □ Neutral, □ Agree, □ Strongly Agree
b. Guided endodontics improves the predictability of access cavity preparation in calcified cases	□ Strongly Disagree, □ Disagree, □ Neutral, □ Agree, □ Strongly Agree
c. Guided endodontics helps preserve tooth structure compared to conventional techniques	□ Strongly Disagree, □ Disagree, □ Neutral, □ Agree, □ Strongly Agree
d. Guided endodontics reduces the risk of iatrogenic complications (e.g., perforations)	□ Strongly Disagree, □ Disagree, □ Neutral, □ Agree, □ Strongly Agree
Q5. Frequency of use and case selection	
a. I routinely use guided endodontics for calcified canals in my practice	□ Strongly Disagree, □ Disagree, □ Neutral, □ Agree, □ Strongly Agree
b. I prefer guided endodontics over conventional techniques for severely calcified canals	□ Strongly Disagree, □ Disagree, □ Neutral, □ Agree, □ Strongly Agree
c. I use guided endodontics primarily for anterior teeth with calcified canals	□ Strongly Disagree, □ Disagree, □ Neutral, □ Agree, □ Strongly Agree
d. I use guided endodontics for both anterior and posterior calcified canals	□ Strongly Disagree, □ Disagree, □ Neutral, □ Agree, □ Strongly Agree
Q6. Diagnostic and planning approach	
a. I routinely use CBCT for diagnosing and planning calcified canal cases	□ Strongly Disagree, □ Disagree, □ Neutral, □ Agree, □ Strongly Agree
b. I find CBCT essential for guided endodontic treatment planning	□ Strongly Disagree, □ Disagree, □ Neutral, □ Agree, □ Strongly Agree
c. I am comfortable with the digital workflow (CBCT → STL → guide design → 3D printing)	□ Strongly Disagree, □ Disagree, □ Neutral, □ Agree, □ Strongly Agree
Q7. Perceived clinical outcomes	
a. Guided endodontics has improved my success rates in calcified canal cases	□ Strongly Disagree, □ Disagree, □ Neutral, □ Agree, □ Strongly Agree
b. Guided endodontics has reduced my procedural time for calcified canals	□ Strongly Disagree, □ Disagree, □ Neutral, □ Agree, □ Strongly Agree
c. Patient outcomes have been better with guided endodontics compared to conventional approaches	□ Strongly Disagree, □ Disagree, □ Neutral, □ Agree, □ Strongly Agree
SECTION C: Perceived barriers to adoption.	
Q8. Financial and infrastructural barriers	
a. The cost of CBCT acquisition/access is a significant barrier	□ Strongly Disagree, □ Disagree, □ Neutral, □ Agree, □ Strongly Agree
b. The cost of guided endodontic software and 3D printing equipment is prohibitive	□ Strongly Disagree, □ Disagree, □ Neutral, □ Agree, □ Strongly Agree
c. The cost per case for guide fabrication is too high for routine use	□ Strongly Disagree, □ Disagree, □ Neutral, □ Agree, □ Strongly Agree
d. My practice lacks the necessary infrastructure (CBCT, 3D printer, software) for guided endodontics	□ Strongly Disagree, □ Disagree, □ Neutral, □ Agree, □ Strongly Agree
Q9. Training and technical barriers	
a. Lack of formal training in guided endodontics is a barrier	□ Strongly Disagree, □ Disagree, □ Neutral, □ Agree, □ Strongly Agree
b. The learning curve for digital planning and guide design is steep	□ Strongly Disagree, □ Disagree, □ Neutral, □ Agree, □ Strongly Agree
c. The additional steps (CBCT, planning, printing) increase overall treatment time	□ Strongly Disagree, □ Disagree, □ Neutral, □ Agree, □ Strongly Agree
d. I lack confidence in my ability to perform guided endodontic procedures	□ Strongly Disagree, □ Disagree, □ Neutral, □ Agree, □ Strongly Agree
Q10. Clinical and workflow barriers	
a. Limited applicability in posterior teeth with restricted access is a barrier	□ Strongly Disagree, □ Disagree, □ Neutral, □ Agree, □ Strongly Agree
b. Guide stability and fitting issues are a concern in clinical practice	□ Strongly Disagree, □ Disagree, □ Neutral, □ Agree, □ Strongly Agree
c. The technique is too time-consuming for routine clinical workflow	□ Strongly Disagree, □ Disagree, □ Neutral, □ Agree, □ Strongly Agree
d. I refer calcified canal cases rather than managing them with guided endodontics	□ Strongly Disagree, □ Disagree, □ Neutral, □ Agree, □ Strongly Agree
SECTION D: Solutions and future perspectives.	
Q11. Potential solutions to overcome barriers	
a. Increased availability of affordable CBCT and 3D printing would encourage adoption	□ Strongly Disagree, □ Disagree, □ Neutral, □ Agree, □ Strongly Agree
b. More hands-on training courses and workshops are needed	□ Strongly Disagree, □ Disagree, □ Neutral, □ Agree, □ Strongly Agree
c. Integration of guided endodontics into postgraduate endodontic curricula is essential	□ Strongly Disagree, □ Disagree, □ Neutral, □ Agree, □ Strongly Agree
d. Development of more user-friendly software and streamlined workflows would help	□ Strongly Disagree, □ Disagree, □ Neutral, □ Agree, □ Strongly Agree
e. Insurance coverage or institutional support for guided endodontic procedures would facilitate adoption	□ Strongly Disagree, □ Disagree, □ Neutral, □ Agree, □ Strongly Agree
Q12. Future outlook	
a. I believe guided endodontics will become the standard of care for calcified canals in the next 5–10 years	□ Strongly Disagree, □ Disagree, □ Neutral, □ Agree, □ Strongly Agree
b. I plan to adopt or increase my use of guided endodontics in the near future	□ Strongly Disagree, □ Disagree, □ Neutral, □ Agree, □ Strongly Agree
c. Technologies like AI-assisted planning and augmented reality will further enhance guided endodontics	□ Strongly Disagree, □ Disagree, □ Neutral, □ Agree, □ Strongly Agree
d. I would recommend guided endodontics to colleagues for managing calcified canals	□ Strongly Disagree, □ Disagree, □ Neutral, □ Agree, □ Strongly Agree
